# A Tale of Two Countries—the United States and Japan: Are Differences in Health Due to Differences in Overweight?

**DOI:** 10.2188/jea.JE2008012

**Published:** 2008-12-17

**Authors:** Sandra L. Reynolds, Aaron Hagedorn, Jihye Yeom, Yasuhiko Saito, Eise Yokoyama, Eileen M. Crimmins

**Affiliations:** 1 University of South Florida, Tampa, FL, USA; 2 University of Southern California, Los Angeles, CA, USA; 3 Nihon University, Tokyo, Japan

**Keywords:** cross-national studies, obesity, disease, chronic conditions

## Abstract

**Background:**

Despite similar standards of living and health care systems for older persons, there are marked differences in the relative health of the elderly populations in the United States (US) and Japan. We explore the association of overweight and obesity with these health disparities.

**Methods:**

Data on older adults from the US National Health Interview Survey (1994) and the Longitudinal Study of Aging II (1994) were compared to similar data from the 1999-2001 Nihon University Japanese Longitudinal Study of Aging. Regression analyses for the 2 countries were conducted to examine the correlates of being overweight and obese, and the relationships of overweight and obesity with activities of daily living functioning, heart disease, arthritis, and diabetes.

**Results:**

The prevalence of overweight and obesity is higher in the US than in Japan, as is the prevalence of heart disease, diabetes, arthritis, and functioning problems. Education level and marital status are predictors of overweight for older Americans but not for older Japanese people. Health behaviors affect weight in all groups. The prevalence of functioning problems and disease are more likely to be associated with being overweight in US men and women than in Japanese women, and are not associated with being overweight in Japanese men.

**Conclusion:**

Despite similar standards of living and health care systems for older persons, the conditions associated with poor health differ in the US and Japan. Being overweight or obese appears to be related to more functioning problems and arthritis in the US than in Japan.

## INTRODUCTION

Why do 2 of the most affluent countries in the world-the United States (US) and Japan-differ markedly in terms of health and life expectancy for older adults? Japan has both the highest life expectancy at birth and active life expectancy in the world,^1^^-^^3^ while the US does not even rank among the top 10 countries with regard to the average life span or active life, although life expectancy at the age of 65 is similar between the 2 countries.^2^^,^^4^^,^^5^ This article has 2 objectives: The first objective is to compare the impact that various health behaviors have on the prevalence of overweight in the US and Japan. The second objective is to examine the consequences of overweight on mortality and morbidity in the 2 countries.

Weight may have a particularly strong influence on the relatively poor health of people in the US and the good health of the Japanese. Weight is strongly linked to cultural factors such as diet and activity level, which differ markedly between people in the US and Japan. Given the association between overweight and obesity and health,^6^^,^^7^ and the differences in health and life expectancy between people in Japan and the US, understanding the factors associated with overweight and its consequences in both the US and Japan can help us understand the relative health status of the 2 countries as well as how health differentials within the 2 countries may be differently determined.

By understanding the role played by obesity in health problems in the older populations of Japan and the US as well as the factors associated with obesity in the 2 countries, we should improve our understanding of this serious international public health issue as well as the determinants of differential health in the 2 countries. In this paper, we examine the difference in the likelihood of being overweight and obese by using 2 large nationally representative surveys designed to compare the older populations of the US and Japan. We also examine the association between weight and demographic, lifestyle, and socioeconomic factors and between weight and functioning problems and disease presence in the US and Japan. All these analyses were performed separately for men and women because of the evidence for sex-based differences in both the obesity levels and correlates of obesity.^8^^-^^20^

## METHODS

### Data

Both surveys used in this analysis are designed to provide nationally representative samples of the older population. This study used data from the Nihon University Japanese Longitudinal Study of Aging (NUJLSOA), which was conducted by the Nihon University Center for Information Networking as one of their research projects. Complete information on the design and methods are published elsewhere and will not be presented here.^11^ The population comprising people aged 75 and above was oversampled, and appropriate weighting systems were applied to ensure that the total sample was nationally representative of those aged 65 and above (n = 4,997). The survey included questions on functioning, the presence of chronic conditions and impairments, health behaviors, and social and demographic characteristics. The NUJLSOA was designed to be comparable to the US Second Longitudinal Study of Aging (LSOA II), which was a supplement to the 1994-95 National Health Interview Surveys (NHIS).^12^

Both the 1994-5 NHIS and LSOA II are used in this analysis for the US. The NHIS is an annual survey that is designed to monitor the health of the community-dwelling population of the US. The 1994 sample consisted of 14,571 respondents who were ≥65. Information on the methods and study design of the NHIS surveys has been published elsewhere and will not be presented here.^13^ The LSOA II contains information from a panel of 9,477 respondents aged 70 and above, obtained from the 1994-5 NHIS, which reflects the US noninstitutionalized population aged 70 and above. Sample collection procedures and data description for the LSOA II have also been published elsewhere and will not be presented here.^12^ For comparison with the NUJLSOA 65+ sample, we used the 1994 NHIS to address the prevalence of overweight and obesity for the 65+ US population. For examining the determinants and consequences of overweight and obesity, we used data from respondents in the LSOA II (age, 70 and above) as well as from those in the NUJLSOA, who are also aged 70 and above, in order to examine the relationship of health behaviors and disease presence with the likelihood of being overweight or obese, and the relationships between weight and health outcomes, since the latter are not available in the NHIS.^11^^-^^13^

### Measurements

We first compare the prevalence of overweight and obesity in older Americans and older Japanese adults, using the current World Health Organization (WHO) standards, defining overweight by a body mass index (BMI) of 25.0-29.9 and obesity by a BMI of 30.0 and above.^14^ The BMI was computed using self-reports of weight and height available in both surveys.

Some researchers have suggested that different definitions of overweight and obesity should be used in the US and Japan because studies on the Japanese suggest that they have a higher percentage of body fat at lower BMI levels than Americans or Europeans.^14^ Therefore, we also conducted analyses to examine the effect of being in the highest quintile of weight in Japan (BMI, >24.1) and the US (BMI, >28.5).^14^^,^^15^ The results were robust, so we present the results by using the WHO categories, which are the most widely recognized and most frequently used categories in studies of overweight and obesity, since they put people at a risk for mortality and morbidity. Moreover, using the same definitions for the 2 countries makes the results more comparable.

In addition to weight, we examined 2 additional obesity and health-related behaviors. Smoking was defined as “being a current smoker,” and heavy drinking was defined as consumption of 3+ drinks per day. Although physical activity measures are available in both the US samples and in the Japanese samples, the questions are posed differently, making inter-country comparisons of physical activity problematic. However, considering that physical activity is usually included in studies of excess weight, we conducted the analyses with and without the physical activity measures and found that the results showed little change. Therefore, we present our results without the physical activity measures.

The sociodemographic variables include age (continuous), marital status (married = 1; not married = 0), and urban/rural status. While “urban” in the US is defined as living in or near a relatively large city (with a population of at least 150,000, a metropolitan statistical area in the US), in Japan, it is defined as living outside a rural or fishing village. This definitely makes a comparison of urban/rural status between the 2 countries problematic.

Information on the socioeconomic status (SES) -- e.g., education -- is available from both surveys. In the US survey, the respondents were asked to indicate the total number of years of formal education completed. In the Japanese survey, education was reported with the use of 7 categories in the first wave and by single years in the second wave, collected in 2001. We use the Wave 2 years of education when available, and where the Wave 2 question was not answered (n = 1,041), the median value of the years of education for the respondents in the Wave 1 category was assigned.

Japanese income was converted into US dollars on the basis of the exchange rate as on November 15, 1999. In both the NHIS and NUJLSOA, the household income is reported in categories, and the categorical midpoint is assigned. The number and range of the income categories differ; the US has 50 categories with top-coding at $50,000, and Japan has 13 categories with top-coding at $150,000. We tested the sensitivity of the results to the range of income categories by collapsing the Japanese top categories to match the top US category, and the results were robust; therefore, we used the available details on income. In addition, we included respondents for whom the income information was missing in the regression, by including this as a control variable and setting them at the mean. This is done so that the number of cases in the analysis is not reduced.

The health outcomes investigated include the presence of functioning problems, which is indicated by reporting difficulty in at least one activity of daily living (ADL): dressing, eating, toileting, walking, bathing, and transferring in or out of bed. The questions on functioning in the 2 surveys were designed to be similar and assumed the format “do you find it difficult to [bathe] due to your health or physical state” in Japan, and “because of a health or physical problem, do you have any problem [bathing]” in the US. Chronic conditions were determined on the basis of self-reports of whether a doctor has ever told the respondent that he/she has a heart disease, arthritis, or diabetes in the LSOA II; in the NUJLSOA, the question was worded as “have you ever had or ever experienced the condition?”

### Analysis

We used multinomial logistic regression to examine the correlates of being overweight and obese relative to having lower weight for American men and women and Japanese women. For Japanese men, we included all those who were overweight and obese in one category because there were only 7 obese Japanese men who were 70 or over. For this group, we ran a binary logistic regression, where the dependent variable was having a BMI of ≥25. We then used binary logistic regression to relate being overweight and obese as independent variables (again with the exception of Japanese men, where the category was overweight) to having functioning problems, heart disease, diabetes, or arthritis. These regressions include a variety of sociodemographic factors, including age, gender, marital status, education, income, and urban/rural residence. We also included the states of being a current smoker and a heavy drinker. All regressions were run for Japan and for the US, for males and females, separately. We also pooled the data between countries to test for interactions between the countries and healthy behaviors. Few of the interactions were significant, so we have not presented those results here. Finally, we used the coefficients resulting from these equations to estimate the probability of an overweight, obese, or lower weight person in each country having each of these diseases.

## RESULTS

The characteristics of men and women aged 65 years and above in the 2 samples are shown in Table 1, along with the significance of the differences between the 2 countries. In both samples, the average age of men is approximately 73, and the average age of women is approximately 74. Men in both countries are most likely to be married, while the majority of women are not married. However, Japanese persons of both sexes are more likely to be married than Americans. In both countries, 38% of the people in each group live in non-urban areas.

**Table 1.** Sociodemographic characteristics of older adults in the United States and Japan

	Males (65+)			Females (65+)		

	United States	Japan	*P*	United States	Japan	*P*
Mean age	73.4	72.8	0.0006	74.5	74.1	0.004
% Male	41.7	43.9	0	na	na	
% Married	78	87.1	0.0001	41.6	45.4	0.0005
% Non-urban	37.6	37.8	0.8681	37.9	38.1	0.8804
Mean income 1999, US $	30,540	32,234	0.009	24,641	22,404	0.0001
Mean years of education	11.5	9.5	0.0001	11.1	8.5	0.0001
n	5,969	2,114		8,602	2,883	

Sources: Japan-Nihon University Japanese Longitudinal Study of Aging

United States-65+ 1994 National Health Interview Survey

Older Japanese men live in households with higher mean incomes than those of American men and women and Japanese women. Older Japanese women live in households with lower mean incomes than Americans, resulting in a larger sex differential in the income in Japan than in the US. Japanese older adults of both sexes have a lower level of education than older Americans, and there is also a wider differential in education between the sexes in Japan.

### Prevalence of Overweight and Health Problems

Older Americans are much more likely to be overweight than older Japanese persons (Table 2). We found that 10.5% of the men and 14.5% of the women in the US are obese; in contrast, obesity is almost nonexistent among older persons in Japan: only 0.9% of Japanese men and 2.3% of Japanese women are obese. As indicated above, this low prevalence of obesity in Japanese males is the reason why we combined overweight and obesity into 1 category—overweight (BMI, ≥25)—for the regression analyses.

**Table 2.** Overweight and obese (%) among older adults (65+) in the United States and Japan

Weight Status		Males			Females		

		United States	Japan	*P*	United States	Japan	*P*
Overweight	25 < BMI < 30	51.6	16	0.0001	43.1	20.2	0.0001
Obesity	BMI, 30+	10.5	0.9	0.0001	14.5	2.3	0.0001
Overweight or obese	BMI, 25+	62.1	16.9	0.0001	57.6	22.5	0.0001
n		5,969	2,114		8,602	2,883	

Sources: Japan-Nihon University Japanese Longitudinal Study of Aging

United States-65+ 1994 National Health Interview Survey

More than half of the older men in the US (51.6%) and 43.1% of the women are overweight. In Japan, only 16.0% of older men and 20.2% of the women are overweight. Therefore, the sex-based differences are different in both countries. In Japan, women are more likely to be both obese and overweight. In the US, it is men who are more likely to be overweight and women who are more likely to be obese.

Health behaviors are not always better in Japan than in the US, as Japanese men report slightly higher rates of heavy drinking than Americans (Table 3). The rates of smoking are considerably higher among Japanese men than among US men, and are the lowest among Japanese women.

**Table 3.** Prevalence of self-reported functioning difficulty, disease, and health behaviors in the US and Japan in people aged 70+

	Males			Females		

	United States	Japan	*P*	United States	Japan	*P*
% Heavy drinkers	11.6	14.4	0.0001	3.3	0.8	0.0001
% Current smokers	11.1	32.8	0.0001	9.2	6	0.0001
% with ADL difficulty	23.3	8.5	0.0001	31.3	11.3	0.0001
% with arthritis	49.5	10.3	0.0001	63.8	19.7	0.0001
% with heart disease	24.7	16.2	0.0001	19.2	16.4	0.0021
% with diabetes	12.9	10.6	0.01	11.5	8.6	0.0001
n	3,744	1,532		5,703	2,301	

Sources: Japan-Nihon University Japanese Longitudinal Study of Aging

United States-70+ Longitudinal Study of Aging II

All 3 diseases and functioning problems are more prevalent in the US than in Japan. The prevalence of ADL functioning difficulties is approximately 3 times as high for older adults in the US compared to those in Japan. Compared to the prevalence in Japan, arthritis is approximately 3 times as prevalent among American women and 5 times as prevalent among American men. The reported prevalence of both heart disease and diabetes are significantly higher in the US than in Japan.

### Correlates of Overweight

Table 4 presents the results of multinomial logistic regression, which addresses how sociodemographic and lifestyle factors relate to overweight and obesity in the 2 countries. Increasing age is associated with a lower likelihood of being overweight and obese in both countries and for both genders. Each year of age is associated with a 6-9% reduction in the likelihood of being overweight, and a reduction of approximately 8-9% in the likelihood of being obese.

**Table 4.** Odds ratios (lower, upper confidence intervals) from multinomial logistic regression for factors related to being overweight (BMI, 25.0-29.9) or obese (BMI, 30+)^a^: 70+ individuals in Japan and the United States

	Males				Females			

	United States		Japan^b^		United States		Japan	

Overweight	Odds ratios	Lower, upper CI	Odds ratios	Lower, upper CI	Odds ratios	Lower, upper CI	Odds ratios	Lower, upper CI
Age	0.91^‡^	0.90, 0.93	0.94^†^	0.90, 0.97	0.94^‡^	0.93, 0.95	0.93^‡^	0.90, 0.95
Education	0.98*	0.96, 0.99	1.02	0.96, 1.08	0.93^‡^	0.92, 0.95	0.98	0.93, 1.03
Income	0.99*	0.99, 0.99	1.01	0.99, 1.01	0.99	0.99, 1.00	0.99	0.99, 1.01
Married	1.21*	1.03, 1.43	0.89	0.56, 1.42	0.93	0.82, 1.05	0.87	0.66, 1.14
Non-urban	1.09	0.93, 1.27	0.85	0.60, 1.23	1.03	0.90, 1.17	0.82	0.63, 1.07
Current smoker	0.40^‡^	0.32, 0.50	0.62*	0.42, 0.92	0.50^‡^	0.41, 0.62	1.07	0.63, 1.82
Heavy drinker	1.56^†^	1.16, 2.11	1.69*	1.09, 2.62	1.05	0.62, 1.77	1.74	0.41, 1.74
-2LL (X^2^)	4672.44	252.8^‡^	969.82	33.5^‡^	6886.56	222.8^‡^	1604.42	33.3^‡^
n	3,592		1,404		5,415		2,073	

Obese								

Age	0.92^‡^	0.89, 0.94			0.92^‡^	0.91, 0.94	0.90*	0.83, 0.98
Education	0.94^‡^	0.91, 0.97			0.91^‡^	0.89, 0.93	0.95	0.82, 1.11
Income	0.99*	0.98, 0.99			0.99	0.99, 1.00	0.99	0.96, 1.02
Married	0.84	0.65, 1.09			0.79*	0.67, 0.94	1.11	0.53, 2.33
Non-urban	1.13	0.89, 1.44			1.03	0.86, 1.23	0.74	0.35, 1.56
Current smoker	0.27^‡^	0.17, 0.45			0.44^‡^	0.32, 0.61	0.63	0.11, 3.52
Heavy drinker	1.52*	1.01, 2.30			1.37	0.70, 2.70	0.00	0.00, 0.00
-2LL (X^2^)	2284.85	108.2^‡^			4084.82	183.9^‡^	323.86	11.0
n	3,592				5,415		2,073	

* *P* < 0.05, † *P* < 0.01, ‡ *P* < 0.001

^a^ Reference category = BMI values less than 25; models controlled for missing income.

^b^ Overweight and obese

Sources: Japan: Nihon University Japanese Longitudinal Study of Aging

United States: Longitudinal Study of Aging II

Socioeconomic influences result in some differences between the 2 countries. American men and women with higher education are less likely to be overweight or obese. Each year of education reduces the relative likelihood by 2-9%. Overweight or obesity is also observed to a lower extent in men with higher incomes. In Japan, there is no evidence of a link between the SES and weight. Moreover, marriage is not associated with weight in Japan. Among Americans, the association differs according to gender. Married men are 21% more likely to be overweight; in contrast, married women are 21% less likely to be obese. Urban residence is not associated with weight in either country.

Healthy behaviors are also responsible for the differences between the 4 groups. Being a current smoker is associated with a 37-60% lower likelihood of being overweight in all but Japanese women, among whom smokers are quite rare. It is associated with an even lower relative likelihood of obesity for American men and women. Finally, being a heavy drinker is related to an increased likelihood of being overweight and obese among US men and being overweight among Japanese men. Among women, there is no significant relationship.

### Health Outcomes Associated with Overweight and Obesity

In Table 5, we address the question of whether weight is associated with the presence of disease and functioning problems in the same manner in the 4 groups. The results indicate the odds ratios—resulting from a set of 4 binary logistic regressions for each country and gender group—of the probability of having self-reported ADL difficulty, arthritis, diabetes, or heart disease based on the overweight and obesity status. Each regression includes controls for other variables that are likely to affect the outcomes including age, education, income, missing income, marital status, non-urban residence, smoking status, and heavy drinking. Again, for Japanese men, only the prevalence of overweight has been considered. Before going into the details of the results, 3 points can be made. The first is that being overweight is not associated with a higher likelihood of any of these health problems among Japanese men. The second is that heart disease is not significantly more common among the obese or overweight in any of the 4 groups. The third is that the other 3 conditions-ADL difficulty, arthritis, and diabetes-are more likely to occur with higher weight in American men and women and Japanese women.

**Table 5.** Odds ratios (lower, upper confidence intervals) for effects of obesity or overweight on self-reported functioning difficulty and disease presence^a^ among american and Japanese adults 70 and older

	US males				Japanese males^c^			

	Odds ratio	Lower, upper CI	Adj. R^2^	H-L test ^b^	Odds ratio	Lower, upper CI	Adj. R^2^	H-L test ^b^
ADL difficulty								
Obese	1.608^‡^	1.234, 2.095	0.0645	0.847				
Overweight	0.867	0.726, 1.035			0.637	0.340, 1.194	0.075	0.2775
Arthritis								
Obese	1.533^‡^	1.211, 1.941	0.0364	0.6949				
Overweight	1.350^‡^	1.167, 1.562			1.465	0.904, 2.374	0.0261	0.572
Diabetes								
Obese	1.464^‡^	1.096, 1.955	0.0402	0.1271				
Overweight	1.474^‡^	1.180, 1.842			0.574	0.307, 1.072	0.0371	0.3866
Heart disease								
Obese	1.152	0.890, 1.491	0.0137	0.4767				
Overweight	0.965	0.816, 1.142			1.401	0.941, 2.807	0.0213	0.3196


	US females				Japanese females			

	Odds ratio	Lower, upper CI	Adj. R^2^	H-L test ^b^	Odds ratio	Lower, upper CI	Adj. R^2^	H-L test ^b^

ADL difficulty								
Obese	2.085^‡^	1.730, 2.514	0.0985	0.8695	2.965*	1.153, 7.628	0.1248	0.8009
Overweight	1.292^†^	1.121, 1.490			0.963	0.639, 1.542		
Arthritis								
Obese	1.572^‡^	1.282, 1.928	0.0416	0.9868	2.686†	1.288, 5.601	0.0313	0.7285
Overweight	1.624^‡^	1.423, 1.853			1.542†	1.138, 2.089		
Diabetes								
Obese	1.802^‡^	1.434, 2.265	0.0707	0.416	1.187	0.403, 3.495	0.0332	0.1857
Overweight	1.742^‡^	1.419, 2.139			1.494	0.977, 2.285		
Heart disease								
Obese	1.16	0.933, 1.443	0.0244	0.5546	1.009	0.386, 2.638	0.0317	0.5583
Overweight	1.084	0.923, 1.273			1.052	0.745, 1.486		

* *P* < 0.05 † *P* < 0.01 ‡ *P* < 0.001

^a^ Controlled for age, education, income, missing income, marital status, non-urban residence, smoking, and drinking

^b^ H-L: Hosmer-Lemeshow test of model fit

^c^ Overweight and obese are combined

Sources: Japan-Nihon University Japanese Longitudinal Study of Aging

United States-Longitudinal Study of Aging II

Being obese significantly increases the likelihood of having difficulty in performing ADLs for Americans of both sexes and Japanese women. The relative extent of the effect is greater in women (OR, 2.1 for US females and 2.9 for Japanese females) than in US men (OR, 1.6). In American women, being overweight is also associated with an increase in the likelihood of functioning problems. Being obese increases the likelihood of having arthritis in Americans by 57-61% and is associated with a 3-fold higher risk of arthritis in Japanese women. In each of these groups, being overweight is associated with an increase of 35-62%. Diabetes is 46% more likely to be prevalent among obese American males and 80% more likely to be prevalent among obese American women. For American men and women only, being overweight increases the relative likelihood of diabetes by 47-74%.

In order to clarify the association of weight with health conditions, we used the results of the equations in Table 5 to predict the probability that men and women in the US and women in Japan with similar characteristics will have each of the individual health problems. We attempt to estimate the probability of an obese and overweight person having ADL difficulty, arthritis, diabetes, and heart disease, assuming that the person is a married 70-year-old urban dweller with 12 years of education and an average income and is a not a smoker or a heavy drinker. We used all the model coefficients, even if the variable was not significant, in calculating the predicted presence of health problems. We did not conduct this analysis in Japanese men because of the lack of relationships, as can be seen in Table 5. Our results show that obese and overweight Japanese women and American men and women with the same characteristics would have almost identical levels of diabetes and heart disease severity; however, Americans in every weight category would be more likely to have arthritis and functioning difficulty.

## DISCUSSION

In this article, we examine the correlates and consequences of overweight and obesity in the US and Japan in an attempt to gain insight into the role of weight on health in these 2 highly developed countries.

### Overweight and Obesity in the US and Japan

The prevalence of overweight and obesity in the US is relatively high and is increasing, from 15% obese adults in 1980 to 32% obese adults in 2000.^16^ In the US, obesity is more common among persons in their late middle age, women, African Americans, married men, and persons with low education or income.^8^^,^^17^^-^^22^ There appears to be an interaction among these factors, so the relationships differ by a combination of gender, race, and age.^23^ Other related factors are lifestyle behaviors such as a fatty diet, lack of exercise, and excessive drinking, all of which are related to lower SES in the US.^20^^,^^24^

Because of recent increases in weight in the Japanese population, there is increasing research on the health impact of overweight and obesity in Japan, and the Japanese government has recently required companies to begin monitoring the weight status of their employees.^25^^,^^26^ However, there is little evidence of a link between weight and low SES in Japan.^27^^-^^30^ There is some evidence to suggest that the rates of overweight and obesity are higher among women and rural residents in Japan, while higher rates of smoking tend to be associated with lower rates of overweight and obesity.^8^^,^^31^^-^^33^

### Association between Overweight and Health Outcomes

With lower levels of overweight in the Japanese population, it is possible that weight may not play the same role in causing adverse health outcomes as it does in the US population. However, researchers have found strong links between weight and heart disease, as well as hypertension, hyperlipidemia, and diabetes in older Japanese adults.^34^^,^^35^ The consequences of being overweight and obese in the US include heart disease, certain types of cancers, strokes, diabetes, atherosclerosis,^36^ and osteoarthritis.^37^ Being overweight or obese is also associated with inactivity, which results in lower aerobic capacity and less muscle strength,^38^ and greater potential for disability and functioning loss.^7^^,^^39^^,^^40^

Factors such as mortality^9^^,^^42^^,^^43^ and health-related quality of life^40^^,^^44^ indicate that the association between health and weight in both Japan and the US are stronger in young to middle-aged adults than in older adults.^41^ In addition, there is evidence that obesity affects the health of men and women differentially in both the US and Japan.^39^^,^^40^^,^^45^^,^^46^

### Correlates of Overweight and Obesity

Our study suggests a different relationship between some sociodemographic and lifestyle factors and the likelihood of being overweight between the 2 countries. In the US, being married is related to weight, but the relationship is different between women and men. As expected, in the US, lower SES is more strongly related to being overweight. In Japan, the only suggestion of a relationship between SES and being overweight was observed in men, among whom higher SES was related to being overweight.

There are also differences in the influence of some lifestyle factors on being overweight. Heavy drinking is related to being overweight in both US and Japanese men, while in both Japanese men and US men and women, not smoking is associated with a higher likelihood of being overweight.

### Health Outcomes

Our results also indicate the markedly better health of older Japanese persons, i.e., persons aged 70 and above, compared to persons of similar age in the US. The prevalence of functioning problems and arthritis is greater in the US than in Japan. Among both men and women, the levels of heart disease and diabetes are higher in the US than in Japan, but the differences are relatively small compared to the differences in functioning and arthritis.

Our findings suggest that the links between overweight and obesity and heart disease and diabetes are somewhat similar in American men and women and Japanese women. Our estimates of the proportion of people who would have diabetes and heart disease if they had the same behaviors and characteristics is similar for men and women in the US and Japanese women, irrespective of whether they are obese, overweight, or of lower weight. In Japanese men, weight does not appear to be related to this set of health outcomes. This is consistent with the findings of Kadowaki and colleagues,^47^ who verified the presence of higher visceral adipose tissue (VAT) in Japanese men with the same waist circumference as Caucasian men. These findings suggest a potential reason why Japanese men are less likely to be obese but are at a higher risk of metabolic diseases than American men. This almost certainly does not represent genetic differences, as research on Japanese-American men in the Honolulu Heart Program clearly indicates obesity levels that are much closer to those of American men.^48^

The higher prevalence of functioning problems and arthritis in the US are only partly due to differing characteristics and behaviors. Even if Japanese and American women exhibit the same behaviors and characteristics in our model, the rates of ADL difficulty and arthritis are still predicted to be twice as high in the US in each weight category.

The finding of an association between weight and arthritis in both US and Japanese women raises the possibility of a recursive effect of weight and inactivity, in which women with knee arthritis or osteoporosis become less active, which leads to higher rates of overweight and obesity, which in turn lead to more functioning or ADL problems. The high levels of functioning problems and arthritis in the US may also be due to differences in movement and regular activity that we have not been able to obtain in the data available to us. The cross-sectional nature of the data does not allow us to make assumptions about the time order of the inactivity-weight-disability process, which may also be different in older adults than in the general population.

The SES relationship is stronger in the US than in Japan, implying a weaker social gradient in health in Japan than in the US.^27^^,^^42^^,^^44^^,^^50^^-^^55^ Kawachi^30^ suggests that the impact of social capital is less in relatively egalitarian nations. It is possible that the smaller dispersion about the average levels of education in men observed in Japan results in fewer SES-related differences, a generally greater similarity of behavior, and a less consistent pattern of ill health according to occupational status.^45^^,^^53^^,^^56^^-^^58^ The relative disparity in SES in the US compared to Japan almost certainly plays a part in these differences. It is also possible that eating and exercising patterns are associated with SES to a greater extent in the US. These results imply that the relationship between SES and health in Japan may be different from that observed in the US.^42^^,^^44^

**Table 6.** Predicted probabilities of having ADL difficulty, arthritis, diabetes, or heart disease for married urban-dwelling US men and women and Japanese women aged 70 with 12 years of education and an average income, who are not smokers or heavy drinkers

	US men	US women	Japanese women
ADL difficulty			
Obese	0.204	0.29	0.139
Overweight	0.114	0.17	0.075
All the others	0.079	0.148	0.065
Arthritis			
Obese	0.548	0.734	0.347
Overweight	0.43	0.632	0.248
All the others	0.322	0.52	0.172
Diabetes			
Obese	0.227	0.237	0.216
Overweight	0.149	0.157	0.142
All the others	0.092	0.097	0.087
Heart disease			
Obese	0.212	0.203	0.222
Overweight	0.189	0.181	0.197
All the others	0.169	0.161	0.176

## CONCLUSIONS

From these analyses, we conclude that differences in health between Americans and Japanese are partially due to the differences in weight. We observed that people with the same weight in Japan and the US have the same likelihood of having diabetes, and that this probability increases with weight. However, Americans are more likely to have weight levels associated with a higher probability of diabetes.

Some studies have suggested that one reason for relatively good health in Japan is the presence of a well-established system of universal health care.^59^ This argument might have more merit if we were not discussing older adults—the only segment of the US population that receives virtually universal health care. Certainly, differences exist between the US and Japan in terms of the coverage and affordability of health care, but it is doubtful that universal health care or a lack of it could be responsible for all the differences we found, especially the differences in functioning loss and arthritis, which is not a disease that can be eliminated through treatment or a disease that requires an extremely sophisticated medical approach.

This study has some limitations, the main one being its cross-sectional nature, which allows us to observe relationships but does not help us draw assumptions of causality. As with other studies based on large national surveys, our conclusions are dependent on the accuracy of self-reported data and availability of data, as well as the possible problems in matching variables across country surveys.

Although clinical diagnosis of health is almost always preferable to self-reported health conditions, e.g., diabetes, the accuracy of self-reporting has been found to have high specificity and a positive predictive value, albeit with low sensitivity.^60^^,^^61^ Although it has been noted that self-reports of arthritis and diabetes are quite acceptable for use in studies such as this, we acknowledge the fact that self-reported arthritis is not always in agreement with a medical diagnosis.^62^^-^^64^ Heart disease presents a different issue, as its diagnosis by a doctor as well as by a lay person is difficult. In a comparison study, for example, Ni^65^ found that the prevalence of self-reported heart disease in the National Health Interview Survey 1999 was approximately ½ of that found in the clinically determined Framingham Heart Study, although these samples differ. Nevertheless, with regard to clinically diagnosed heart conditions in the US and Japan, there is no basis for assuming that there is a difference in patient awareness.

Many studies have used self-reported diabetes in large-scale surveys for studying the association between diabetes and obesity and between diabetes and heart disease.^66^^,^^67^ Indeed, the Palloni study specifically used self-reports of both diabetes and heart disease to examine health across several countries in Latin America and the US. In addition, Alonso and colleagues (2004) compared the self-reported prevalence of arthritis, hypertension, diabetes, and heart disease in 8 countries (Denmark, France, Germany, Italy, Norway, the Netherlands, Japan, and the US);^68^ in fact, like the 3 surveys used in this study, major surveys in most of these countries have been designed to have measures that are as comparable as possible.

Other potential concerns with self-reported health conditions include the possibility of differences across countries in the diagnostic practice and diagnostic criteria of the chronic diseases in our study, particularly in the case of conditions where symptoms might be unknown to the respondent. However, the diseases selected (diabetes, heart disease, and arthritis) are associated with very well-known symptoms that affect daily functioning and behaviors, and the questions were worded (“has a doctor told you···”) in such a way as to reduce false positives, so we believe this concern to be a relatively minor one.

Another difference between our data and typical epidemiology studies is our use of nationally representative data, whereas epidemiologists tend to collect data based on specific diseases and specific geographical areas. Examples of highly regarded studies of this type include the WHO MONICA Study, the Honolulu Heart Study, the Framingham Study, and the work of Fujimoto and colleagues on various aspects of the metabolic syndrome.^69^^-^^72^ While cross-national interpretations of data are gathered for the purpose of examining highly specific diagnoses, broader classifications of disease, as used in our study, especially those with very clear life-altering symptoms, should be considered in a different light. Furthermore, the US and Japan both provide national health insurance for the age group studied. Thus, participants in both samples should have adequate access to doctors capable of diagnosing the symptoms of these common conditions. While there may be cross-national differences in the threshold of severity at which the diagnosis is considered clinical, our concern is primarily with symptomatic individuals in both the US and Japan. Therefore, we are of the opinion that direct comparisons between epidemiological studies using city-based samples may not be appropriate, as the studies, while methodologically rigorous, lack the nationally representative nature of the NUJLSOA or the LSOA.

Another limitation includes information on diet—one of the major cultural differences related to weight—which is not available in the surveys. Another issue is the lack of findings for Japanese men, which may be related to their somewhat smaller sample size, as women outlive and outnumber men at these ages.

Trends indicating worsening levels of overweight and obesity may be more important for the US because of the stronger link between obesity and some health outcomes compared to Japan. However, the stronger effects of lifestyle on weight in the US also imply that improvements in health behaviors can have important positive influences on weight and health, even at advanced ages. The implications of this study are that public health officials in Japan, while dealing with a society whose socioeconomic characteristics have relatively little impact on weight or health, may have greater success in implementing public health policies in areas where healthy life behaviors are observed among the general population of older adults. In contrast, the implications for the US are that public health efforts at modifying specific behaviors and meeting health goals may have to be tailored to different subgroups of the population, particularly those of low SES.
